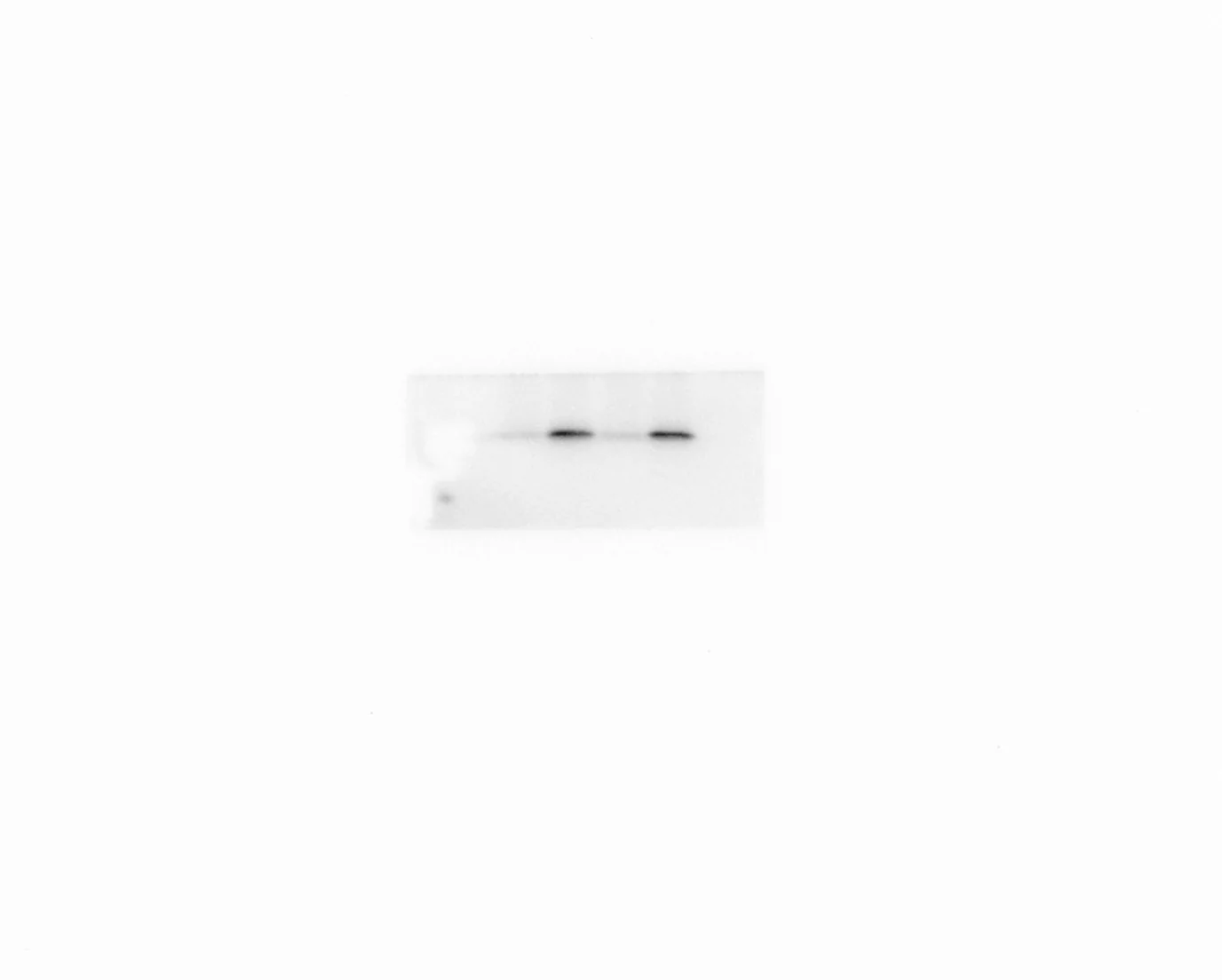# Discernible phenotypic alterations following *linc-10* knockout in *Caenorhabditis elegans*

**DOI:** 10.1016/j.ncrna.2026.10.004

**Published:** 2026-11-04

**Authors:** Chenxi Zhou, Xiaoman Sun, Ruikun Wu, Zifan Wu, He Chen

**Affiliations:** a State Key Laboratory of Opto-Electronic Information Acquisition and Protection Technology, Information Materials and Intelligent Sensing Laboratory of Anhui Province, Anhui University, Hefei, 230601, China; b USTC RNA Institute, School of Basic Medical Sciences, Division of Life Science and Medicine, University of Science and Technology of China (USTC), Hefei, 230027, China

**Keywords:** lncRNAs, Small RNAs, Stress, Phenotype, *Caenorhabditis elegans*

## Abstract

Long noncoding RNAs (lncRNAs) are regulatory molecules that play vital roles in various cellular processes. Although the loss of function of most annotated lncRNAs in several model organisms does not result in severe phenotypes, suggesting they are not essential for survival or reproduction, lncRNAs may still act as responsive elements under diverse physiological and pathological conditions. In this study, we investigated the role of the *linc-10* gene in *Caenorhabditis elegans* and found that its deletion is associated with altered gene expression and impaired reproductive fitness. Knockout (KO) mutants of *linc-10* exhibited mild but notable abnormalities, including reduced brood size, altered developmental trajectories, and increased sensitivity to stress, along with occasional sterility and a slightly elevated male ratio. We characterized the expression patterns of *linc-10* across developmental stages and performed high-throughput sequencing to detect differentially expressed genes (DEGs) following *linc-10* deletion. We found that several immune-related genes were dysregulated. Notably, *linc-10* mutants showed increased susceptibility to *Pseudomonas aeruginosa* PA14 infection and other stressors. Small RNA sequencing further revealed alterations in piRNA and miRNA profiles after *linc-10* deletion. Importantly, CRISPR/Cas9-mediated insertion of a *Plinc-10::linc-10* rescue transgene into a neutral intergenic site partially rescued the reduced brood size and stress-sensitive phenotypes of *linc-10* mutants, supporting the functional relevance of the *linc-10* transcript. Together, these findings suggest that *linc-10* contributes to reproductive fitness, immune homeostasis, and stress resilience in *C. elegans*.

## 1 Introduction

Long noncoding RNAs (lncRNAs) regulate gene expression through diverse mechanisms, including chromatin remodeling, transcriptional control, and post-transcriptional regulation [1], [2]. They act in trans or cis under various physiological and pathological conditions [3]. However, studies on lncRNAs in animal models have raised many unexplained questions. For example, the loss of function of some well-known regulatory lncRNAs does not affect survival and reproduction [4]. Additionally, systematic studies covering nematodes, fruit flies, zebrafish, and mice have demonstrated that the vast majority of lncRNAs are not essential for organismal viability under standard laboratory conditions [5], [6], [7], [8]. Despite this, the dynamic expression of lncRNAs can be altered by a variety of biotic and abiotic stresses, including heavy metals, reactive oxygen species, and pathogenic infections [9], [10]. This raises an important issue in lncRNA biology: the absence of severe phenotypes under standard laboratory conditions does not necessarily indicate a lack of biological function [4]. Many regulatory noncoding RNAs may instead contribute to physiological homeostasis, producing mild or conditional phenotypes that become evident in specific developmental, reproductive, genetic, or environmental contexts [11], [12], [13], [14]. Thus, discernible non-lethal phenotypic alterations can provide important entry points for understanding how lncRNAs support organismal robustness [4], [13].

The functions of lncRNAs in whole animals need to be further elucidated, and *Caenorhabditis elegans* (*C. elegans*) is a suitable research model for this topic [15], [16]. In our previous study, we generated CRISPR-based knockout strains for most long intergenic noncoding RNAs (lincRNAs) in *C. elegans* (155 of 170). Interestingly, fewer than one-fifth of the mutants exhibited alterations in simple physiological indicators [8]. None of the 155 knocked-out lincRNAs resulted in severe phenotypes such as lethality or sterility [4], [8]. One study of highly expressed lincRNAs in the nematode gonad showed that deletion of these lincRNAs did not affect the cell cycle or mitosis, although *linc-4* mutants exhibited a minor growth delay through impaired germline–soma interactions [17]. Another study demonstrated that loss of the lncRNA *lep-5* led to abnormal male tail morphology by affecting LEP-2-mediated LIN-28 degradation and thereby regulating cell differentiation and migration [18]. In addition to these systematic knockout and functional studies, spatiotemporal expression profiling and recent reports have further suggested that *C. elegans* lincRNAs may be associated with developmental regulation, germline-related growth, fertility, male-specific reproductive traits, and environmental responses [19], [20], [21], [22]. These findings suggest that although most lincRNAs are dispensable for viability and basic fertility, mutants with mild but discernible defects may reveal regulatory loci required for maintaining gene expression homeostasis and optimal physiological performance under normal or challenged conditions.

In *C. elegans*, there is a simple but effective innate immune system that distinguishes foreign molecules and defends against other organisms [23]. Important immune signaling pathways, such as the PMK-1/p38 MAPK pathway and the Toll-like receptor pathway, contribute to defense against microbial infections by regulating the expression of antimicrobial peptides (AMPs) and shaping metabolic and behavioral plasticity [24]. Gain or loss of function in genes within these pathways is often associated with alterations in the worm’s physiological traits or phenotypes [24]. Notably, innate immunity and environmental stress responses are closely interconnected, with many signaling pathways participating in both [25]. For example, mutations in the DAF-2/DAF-16 insulin-like signaling pathway can lead to changed responsiveness to both pathogens and abiotic stresses [26]. As regulatory molecules, noncoding RNAs are involved in innate immunity and stress responses [27], [28]. In nematodes, noncoding RNAs, especially small RNAs and their associated transcriptional and regulatory networks, play essential roles not only in cell fate determination and development, but also in responses to biotic and abiotic stresses [29], [30], [31]. These RNA-based regulatory pathways are highly active in germ cells and early embryos; therefore, fertility and germ cell-related phenotypes are often closely associated with stress and infection [32], [33]. Such alterations in germ cell homeostasis, a process closely linked to small RNA pathways including PIWI-interacting RNAs (piRNAs), can lead to phenotypes such as infertility and embryonic lethality [34]. However, whether lncRNAs contribute to immune homeostasis and stress resilience at the whole-organism level remains largely unexplored.

Previously, we identified several functional lincRNAs in *C. elegans*, whose knockout strains exhibited abnormalities in six phenotypic traits [8]. In the present study, we focused on the *linc-10* knockout mutant and observed stress-like phenotypes, including reduced brood size, delayed development, and increased sensitivity to infection and stress. These phenotypes were accompanied by dysregulation of both mRNAs and small RNAs. Importantly, site-specific insertion of a *Plinc-10::linc-10* rescue transgene partially restored selected reproductive and stress-sensitive phenotypes. Taken together, our findings suggest that *linc-10* contributes to reproductive fitness and stress susceptibility in *C. elegans*, accompanied by changes in immune-related gene expression and small RNA profiles.

## 2 Materials and methods

### 2.1 Strains and nematode culture

The *linc-10* deletion strain *linc-10(hzhCR11) II* was constructed and described in our previous work [8]. The wild-type N2 (Bristol) strain was obtained from the Caenorhabditis Genetics Center (CGC). Additional strains used in this study included *prg-1(hzhCR195[gfp::3xflag::prg-1])* I [35] and the *Plinc-10::GFP* reporter strain (HC101), which was generated in this study by CRISPR/Cas9-mediated insertion of a GFP cassette into a neutral intergenic region. The *Plinc-10::linc-10* rescue strain was generated by introducing this rescue transgene into the *linc-10(hzhCR11)* mutant background after CRISPR/Cas9-mediated insertion into the same neutral intergenic region. Unless otherwise indicated, all strains were maintained on nematode growth medium (NGM) plates seeded with *Escherichia coli* OP50 at 20 °C [36].

### 2.2 Brood size, sterility, and male ratio assays

Adult worms were allowed to lay eggs for 4 h at 20 °C after which the adult worms were removed. The eggs were permitted to hatch and grow until they reached the L4 stage. L4 worms were then plated individually and transferred daily from the start of egg laying until egg laying ceased. Viable progeny were counted to determine brood size. Animals that produced no viable progeny were classified as sterile. Male ratio was calculated as the percentage of male progeny among the viable adult offspring. For brood size analysis, 15 worms were examined per replicate, with three independent biological replicates. For sterility and male ratio analysis, 100 worms were examined per replicate, with three independent biological replicates.

### 2.3 Larval growth assay

In this study, we analyzed the growth of larvae by body length profile. Larvae were eluted from NGM culture plates to unseeded NGM plates with M9 buffer every 12 h after hatching and imaged with Axio Scope A1 microscope (Zeiss, Oberkochen, Germany). Body length, defined as the length of the central axis from head to tail, was measured using ImageJ. 10 worms were examined per replicate, with three independent biological replicates.

### 2.4 CRISPR-based knock-in

The CRISPR/Cas9 system was used to construct the *Plinc-10::GFP* reporter strain and the *Plinc-10::linc-10* rescue strain. Both transgenes were inserted into the same neutral intergenic region between *W09C5.5* and *linc-1* on chromosome I using the same pair of sgRNAs. For construction of the *Plinc-10::GFP* reporter strain, N2 adult worms were injected with a mixture of plasmids containing pDD162, which expressed the Cas9 protein (30 ng μL^−1^), sgRNA plasmids (30 ng μL^−1^ each), homologous recombination donor template (30 ng μL^−1^), and the co-injection marker *Pmyo-2::mCherry* (pCFJ90) (15 ng μL^−1^). For construction of the rescue strain, *linc-10(hzhCR11)* mutant adult worms were injected with a similar CRISPR/Cas9 mixture containing a donor template carrying the *linc-10* rescue fragment. The *Plinc-10::GFP* reporter cassette contained GFP driven by an approximately 2-kb upstream promoter region of *linc-10*, followed by the *let-858* 3′UTR. The rescue fragment contained the same approximately 2-kb upstream promoter and the complete 425-bp unspliced *linc-10* genomic sequence, including its annotated UTRs. Approximately 200 F1 worms expressing mCherry were isolated, and F3 animals were screened by PCR genotyping. Correct knock-in events were further validated by Sanger sequencing across the insertion junctions. The newly established strains were crossed back to the corresponding parental strain background. The primers and sgRNAs used for CRISPR-mediated strain construction and genotyping are listed in Table S1.

### 2.5 Fluorescence microscopy imaging and measurement

Worms were placed on agar pads and treated with a 10 mM sodium azide solution for anesthesia. Fluorescent images were captured using microscopy. For quantitative comparisons, images from the same experiment were acquired under identical exposure settings. GFP fluorescence intensity was measured using ImageJ after background subtraction. For whole-animal fluorescence analysis, the total body area was selected as the region of interest; for GFP::3×FLAG::PRG-1 analysis, the germline region was selected for fluorescence quantification.

### 2.6 Western blotting

After being separated on SDS-PAGE gels, proteins from whole worm lysates were transferred to nitrocellulose membranes (PALL Corp., USA). The ECL Western blotting protocol (GE Healthcare, USA) was followed for handling the membranes. Western blots were performed using the following antibodies: HRP-conjugated anti-GFP (1:1000, ThermoFisher Scientific, catalog # MA5-15256-HRP, USA), anti-β-actin (1:1000, ZSGB-Bio, catalog # TA-09, China), and HRP-conjugated secondary antibody (1:10,000, Promega, USA). The manufacturers’ websites provide validation information for the antibodies.

### 2.7 RNA extraction and quality control

Synchronized animals were cultured under the same conditions, and L4 larvae and young adults were collected according to developmental timing and morphological criteria. Total RNA was extracted from the collected worms using TRIzol™ LS Reagent (Invitrogen, USA) following the manufacturer’s instructions with minor modifications. Briefly, synchronized worms were collected from culture plates with M9 buffer and washed three times to remove bacteria. Worm pellets were lysed in TRIzol™ LS Reagent by repeated freeze-thaw cycles at −80 °C followed by vortexing. After phase separation with chloroform, the aqueous phase was collected, and RNA was precipitated with isopropanol. RNA pellets were washed twice with ice-cold 80% ethanol and dissolved in DEPC-treated water. Genomic DNA contamination was removed by DNase I treatment, followed by ethanol precipitation and resuspension in DEPC-treated water. RNA concentration and purity were assessed using a Nano-500 spectrophotometer (AIISHENG, China). RNA integrity was assessed after extraction, and RNA quality was reassessed by the sequencing provider before library preparation. Purified RNA samples were stored at −80 °C until library preparation or qRT-PCR analysis.

### 2.8 mRNA library construction, sequencing, and data analysis

For transcriptome profiling, including mRNA analysis, three independent biological replicates were prepared for each genotype at the L4 larval and young adult stages. Ribosomal RNAs were depleted from total RNA using the Illumina Ribo-Zero Gold kit. Library construction was performed with the NEBNext® Ultra™ RNA Library Prep Kit for Illumina® (NEB, USA) according to the manufacturer’s protocol. Briefly, rRNA-depleted RNA was fragmented, followed by first-strand cDNA synthesis using random hexamer primers [35]. Second-strand synthesis, end repair, A-tailing, adapter ligation, PCR amplification, and library purification were subsequently performed. Libraries were sequenced on an Illumina platform in 150-bp paired-end mode. Raw sequencing reads were converted to FASTQ format using bcl2fastq v1.8.4. Clean reads were aligned to the *C. elegans* reference genome (WBcel235) using STAR v2.7.10b. Gene-level read counts were quantified using FeatureCounts v2.0.1. Differential expression analysis was performed using DESeq2, and differentially expressed genes were identified using thresholds of P value < 0.05 and |log2FoldChange| ≥ 1. Benjamini–Hochberg-adjusted *P* values were also calculated using DESeq2 and are reported alongside the original *P* values in the supplementary differential-expression tables; the figures use the *P*-value-based cutoff specified above. Genes with mean TPM (Transcripts Per Million) values greater than 0 were retained for downstream analysis [37]. The processed mRNA-seq expression and differential-expression data are presented in Table S2.

### 2.9 Quantitative real-time PCR

Worms were subjected to three freeze-thaw cycles at −80 °C, followed by thawing to room temperature. RNA was extracted by submerging the worms in TRIzol™ LS Reagent (Invitrogen). A total of 0.5 μg of RNA was reverse-transcribed into complementary DNA (cDNA) using the GoScript™ Reverse Transcription System (Promega). Quantitative real-time PCR was then conducted according to standard protocols using a PikoReal 96 real-time PCR system (Thermo Scientific) and the GoTaq® qPCR Master Mix (Promega). The 18S rRNA gene was used as an internal normalization control and relative expression levels were calculated using the 2^−ΔΔCt^ method. Each experiment included three independent biological replicates. A list of primers is provided in Table S3.

### 2.10 GO analysis and KEGG pathway enrichment analysis

Differentially expressed genes (DEGs) between wild-type and *linc-10* KO worms were subjected to Gene Ontology (GO) analysis and KEGG pathway enrichment analysis. Functional classification and statistical overrepresentation were performed using the clusterProfiler R package (version 4.8.2) with the GO and KEGG databases. GO terms and KEGG pathways with P value < 0.05 were considered significantly enriched.

### 2.11 Lifespan assays

Lifespan experiments were conducted with minimal modifications to previously described protocols [38]. Briefly, synchronized eggs were plated onto NGM plates and cultured at 20 °C in the dark until reaching the L4 stage. To prevent the development of offspring during the lifespan assessments, L4 worms were transferred to NGM plates supplemented with 50 μM FUdR (Sigma, USA) and 100 μg/mL ampicillin. Lifespan assays were performed at 20 °C, with each experiment repeated three times using 40 worms per plate. Deceased worms were removed from the plates and recorded. Missing worms, whether due to desiccation or crawling off the plate, were accounted for in all statistical analyses.

### 2.12 Survival assay of *C. elegans* exposed to *Pseudomonas aeruginosa* PA14

Ten-centimeter NGM plates were fully seeded with an overnight culture of *Pseudomonas aeruginosa* PA14 and incubated at 37 °C for 18 h, followed by 24 h at room temperature to allow full lawn development and virulence induction. Before introducing worms, the plates were equilibrated to 20 °C. Synchronized L4-stage worms were transferred to PA14-seeded plates for the survival assay. Worm survival was scored every day by gentle prodding. Animals that failed to respond were scored as dead. To avoid interference from progeny production, offspring were removed during scoring when necessary, and only the original experimental animals were included in the survival analysis. Animals that crawled off the plate, ruptured, bagged, or were otherwise missing were censored.

### 2.13 Thermal stress assay

L4 worms were transferred to 10 cm NGM plates seeded with *E. coli* OP50 and pre-equilibrated at 37 °C. Plates were maintained at 37 °C, and worm survival was scored every 2 h by gentle prodding until all animals died. LT_50_ values were calculated using Kaplan–Meier survival analysis.

### 2.14 Oxidative stress assay

L4 worms of different strains were transferred to 3 cm NGM plates and exposed to 20 mM hydrogen peroxide (H_2_O_2_) dissolved in M9 buffer. Plates were incubated at 20 °C. Worm survival was assessed every 10 min by gentle prodding with a platinum wire. Animals that failed to respond were scored as dead.

### 2.15 Small RNA library construction, sequencing, and data analysis

Small RNA libraries were prepared using the QIAseq miRNA Library Kit (QIAGEN, Germany) according to the manufacturer’s instructions and sequenced on an Illumina platform in single-end mode by Novogene Co., Ltd. (Beijing, China). Raw reads were processed using ACGT101-miR v4.2 and custom Perl scripts to remove adapters, low-quality reads, low-complexity sequences, and common structural RNAs, including rRNA, tRNA, snRNA, and snoRNA. The retained clean reads were mapped to miRBase v22.0 and piRBase v3.0 to annotate known miRNAs and piRNAs. Differential expression analysis was performed using DESeq2 v1.46.0, with significance defined as P value < 0.05 and |log2FoldChange| ≥ 1. Benjamini–Hochberg-adjusted *P* values were also calculated using DESeq2 and are reported alongside the original *P* values in the supplementary differential-expression tables; the figures use the *P*-value-based cutoff specified above. The processed small RNA-seq expression and differential-expression data are provided in Table S4.

### 2.16 Small RNA identification and target prediction

Putative target genes of differentially expressed miRNAs and piRNAs were predicted using miRanda (v3.3a). The overlap between predicted target genes and DEGs identified from transcriptome data was visualized using the VennDiagram package (v1.7.3) in R.

### 2.17 Rescue assays

Rescue experiments were performed using wild-type N2, *linc-10(hzhCR11)* mutants, and l*inc-10(hzhCR11)*; *Plinc-10::linc-10* rescue animals. Brood size, sterility, male ratio, larval growth, PA14 infection survival, thermal stress survival, and oxidative stress survival were assessed according to the procedures described above. Each rescue experiment included three independent biological replicates, and all genotypes were assayed in parallel under the same experimental conditions.

### 2.18 Statistical analysis

Statistical analyses were performed using GraphPad Prism (version 10) and R where applicable. Categorical data, including sterility and male ratio, were analyzed using Fisher’s exact test. For comparisons between two groups, continuous data were analyzed using unpaired Student’s t-tests. For rescue assays involving three groups, predefined pairwise comparisons were performed between the rescue strain and wild-type N2 controls, and between the rescue strain and *linc-10* knockout mutants using unpaired Student’s t-tests. Survival curves from lifespan, PA14 infection, thermal stress, and oxidative stress assays were analyzed using Kaplan–Meier survival analysis and compared using the log-rank (Mantel–Cox) test. For sequencing data, statistical thresholds are described in the corresponding sequencing analysis sections. Unless otherwise specified in the figure legends, data are presented as means ± standard deviation (SD) from independent biological replicates. A P value less than 0.05 was considered statistically significant.

## 3 Results

### 3.1 Phenotypic effects of *linc-10* deletion in *C. elegans*

We previously generated a lincRNA knockout (KO) library in *C. elegans* using CRISPR [8], from which the *linc-10* KO strain was obtained; this strain carries a 423-bp deletion spanning from the transcription start site to its locus (Fig. S1A). Quantitative reverse transcription PCR (qRT-PCR) was performed to confirm the absence of *linc-10* transcripts in the KO strain (Fig. S1B). Notably, the *linc-10* KO mutants exhibited approximately 2% sterility and 3% male ratio (Fig. 1A). Among fertile hermaphrodites, brood size was significantly reduced compared with the N2 wild type (Fig. 1B). Additionally, we examined developmental growth by measuring worm length at 12-h intervals from hatching to adulthood. The *linc-10* mutants exhibited a slight but significant delay in larval growth; however, no significant difference in body length was observed at the adult stage compared with the wild type (Fig. 1C). Sterile individuals exhibited abnormal germ cells and embryos, whereas no overt morphological abnormalities were detected in fertile hermaphrodites (Fig. 1D). The source numerical data for sterility, male ratio, brood size, and larval growth measurements are provided in Table S5. In summary, these results indicate that loss of *linc-10* causes mild but discernible reproductive and developmental abnormalities in *C. elegans*, including reduced brood size, occasional sterility, an increased male ratio, and delayed larval growth.

**Fig. 1:**
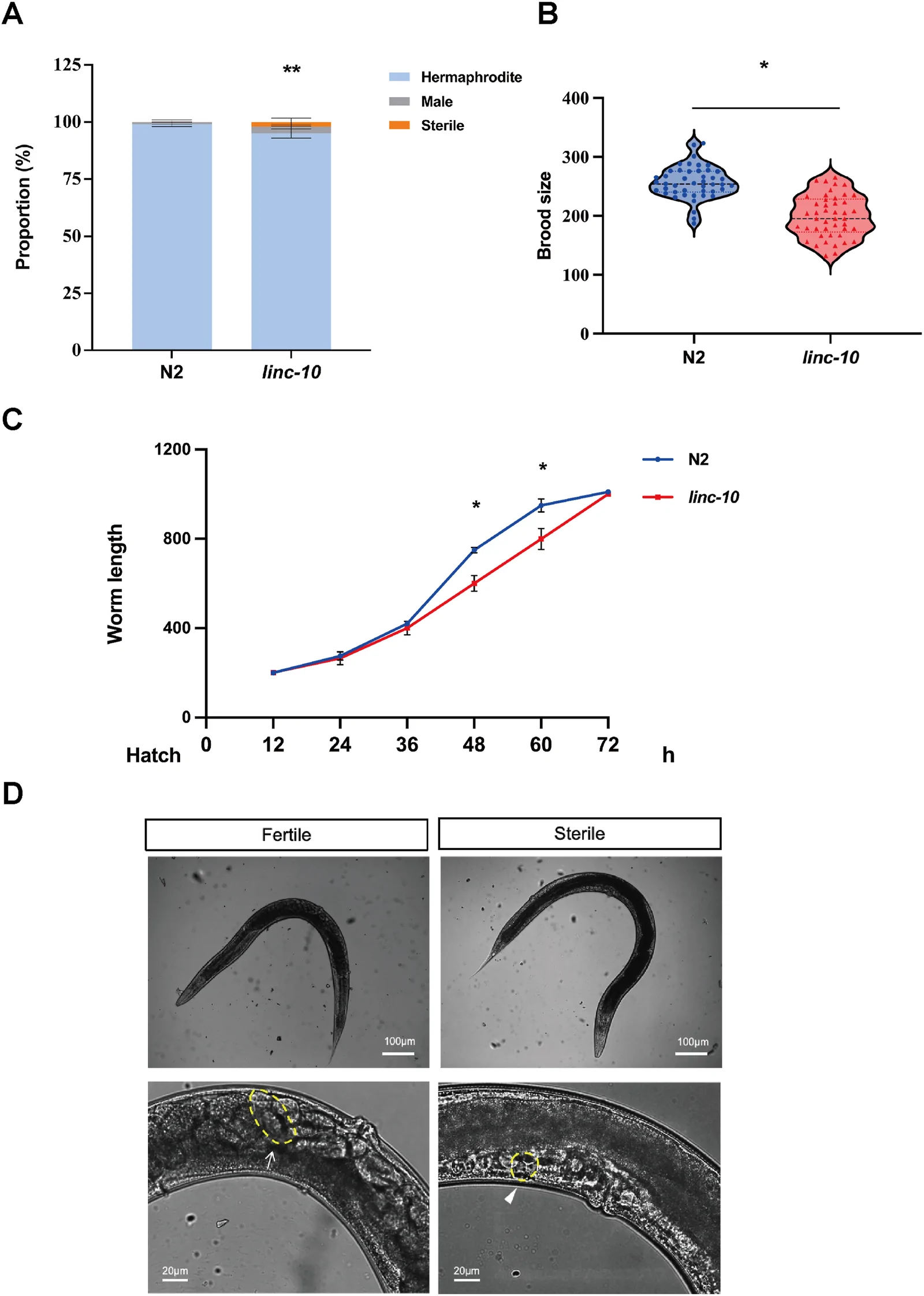
Phenotypic effects of *linc-10* knockout in *C. elegans*. (A) *linc-10* knockout (KO) led to a slight increase in male ratio and the occurrence of sterile individuals. Each independent experiment included 100 worms, and three independent experiments were performed. The overall distribution differed significantly between groups (P = 0.0093). (B) *linc-10* KO resulted in reduced brood size. Fifteen worms were scored in each independent experiment, and three independent experiments were performed. (C) *linc-10* KO altered larval growth. Body length (μm) was measured every 12 h after hatching. Ten worms were measured per time point in each independent experiment, and three independent experiments were performed. (D) Comparison between fertile (left) and sterile (right) *linc-10* KO young adults. White arrows indicate normal embryos, and white arrowheads indicate abnormal or degenerated embryos. Bars represent means ± SD. P < 0.05 (*), P < 0.01 (**), determined by Fisher’s exact test in (A) and unpaired *t*-test elsewhere.

### 3.2 Expression characteristics of *linc-10* at different stages

LncRNAs are generally expressed at lower levels than protein-coding genes, with more diverse transcript profiles across organs, tissues, and cell types, and they also exhibit less conservation across species [1], [4]. RNA-seq data for *C. elegans linc-10* from multiple studies are comprehensively summarized in WormBase [39]. Available RNA-seq data indicate that *linc-10* is detectable across different life stages, with markedly higher expression in males and during the dauer stage than in the classical developmental stages. To further investigate the expression of *linc-10*, we constructed a GFP reporter strain. Using CRISPR, we inserted a GFP coding sequence under the control of the *linc-10* promoter into a neutral genomic locus (Fig. 2A, Fig. S2A). Western blot analysis confirmed the presence of GFP protein in mixed-stage samples of the reporter strain (Fig. S2B). However, fluorescence microscopy revealed that GFP signals in L1 larvae and young adult hermaphrodites were weak and largely indistinguishable from intestinal autofluorescence (Fig. 2B and C). In contrast, distinct GFP signals were detected in the germ cells of males (Fig. 2D). Taken together, these results indicate that *linc-10* expression is generally low under standard developmental conditions but becomes detectable in male germ cells, suggesting a possible involvement of *linc-10* in reproductive processes.

**Fig. 2:**
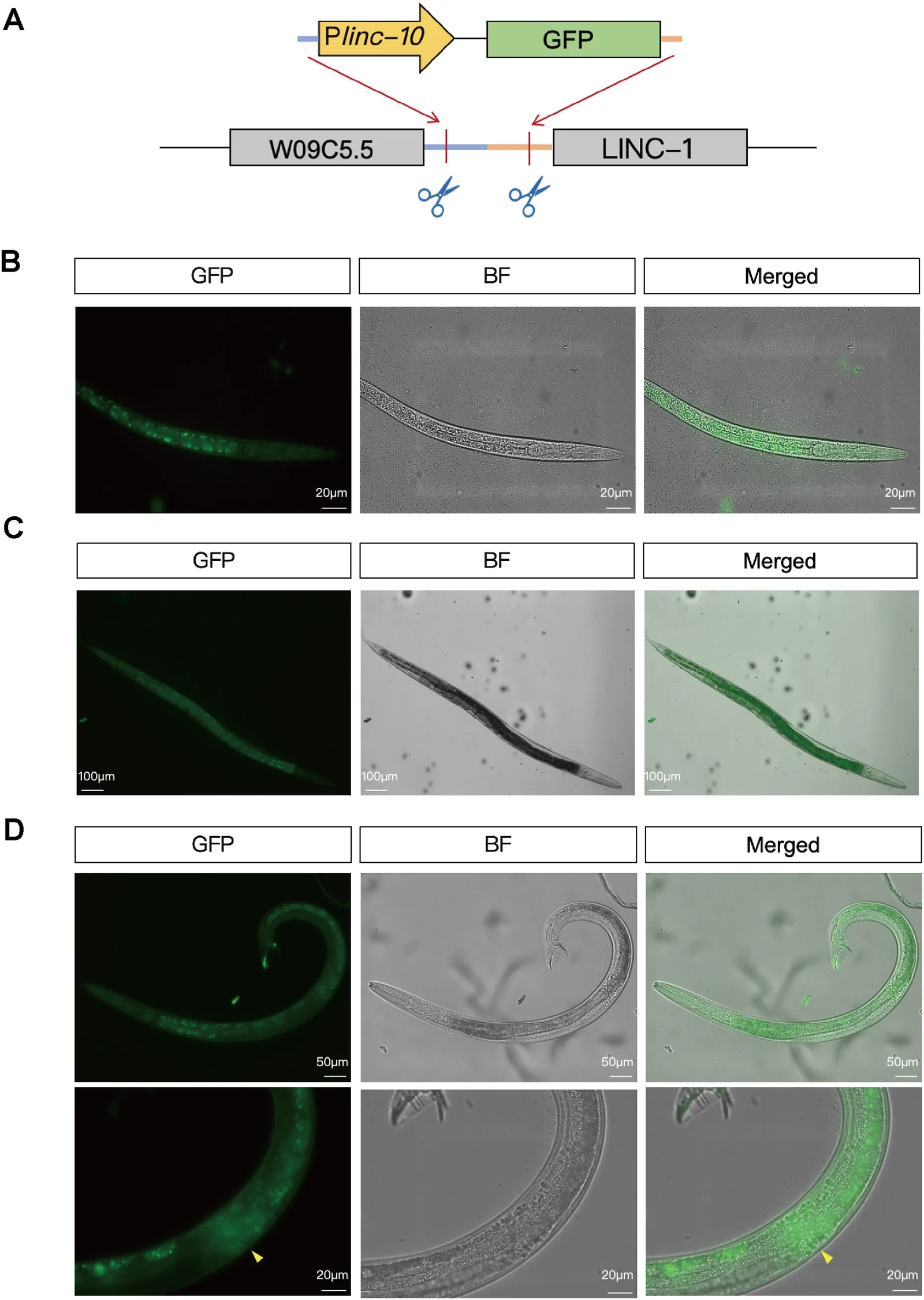
Construction and fluorescence imaging of the *linc-10* reporter strain. (A) A *Plinc-10::GFP* reporter was generated by inserting a GFP cassette under the control of the *linc-10* promoter into a neutral intergenic region between W09C5.5 and LINC-1 on chromosome I using CRISPR/Cas9 genome editing. (B) Fluorescence image of the reporter strain at the L1 larval stage. (C) Fluorescence image of a hermaphrodite adult. (D) In male adults, GFP signal was clearly detected in the male germline (indicated by yellow arrowheads).

### 3.3 Transcriptional profiles of protein-coding genes in the KO strain

Building on these observations, we next examined transcriptional changes in the KO mutants. Gene expression was profiled at the L4 larval and young adult stages, which are critical for germ cell maturation. Principal component analysis (PCA) of the RNA-seq data showed that transcriptional variation was shaped by both developmental stage and genotype (Fig. S3A). In total, 320 protein-coding genes were upregulated and 165 were downregulated in L4 mutants compared with wild-type controls (Fig. 3A, Fig. S3B). At the young adult stage, 816 genes were upregulated and 611 were downregulated (Fig. 3B, Fig. S3C). Among these, 262 genes were consistently altered in both stages (Fig. 3C). Gene Ontology (GO) analysis revealed that differentially expressed genes (DEGs) in the *linc-10* KO strain were enriched in categories related to innate immunity and responses to biotic stress (Fig. 3D and E). KEGG analysis further highlighted pathways associated with stress responses and metabolic processes, including cytochrome P450-mediated metabolism and carbon metabolism (Fig. S3D and E). To validate the RNA-seq results, we examined a subset of immunity-related genes using qRT-PCR, which confirmed the expression changes observed in the sequencing data (Fig. S4, Table S6). Among the dysregulated genes, *prg-1*, which encodes the PIWI Argonaute protein PRG-1 involved in the piRNA pathway and germline homeostasis, was reduced in young adult *linc-10* KO worms. qRT-PCR confirmed the decreased prg-1 transcript level (Fig. S4, Table S6), and GFP fluorescence from the GFP*::*3×FLAG::PRG-1 reporter was also reduced in the germline of *linc-10* KO animals (Fig. 3F–H, Table S7). These results suggest that *linc-10* deletion is associated with reduced PRG-1 expression, although whether this regulation is direct or indirect remains to be determined. Overall, these results indicate that *linc-10* deletion is associated with broad transcriptional changes, particularly in immunity-related and metabolic genes, as well as reduced PRG-1 expression in young adults.

**Fig. 3:**
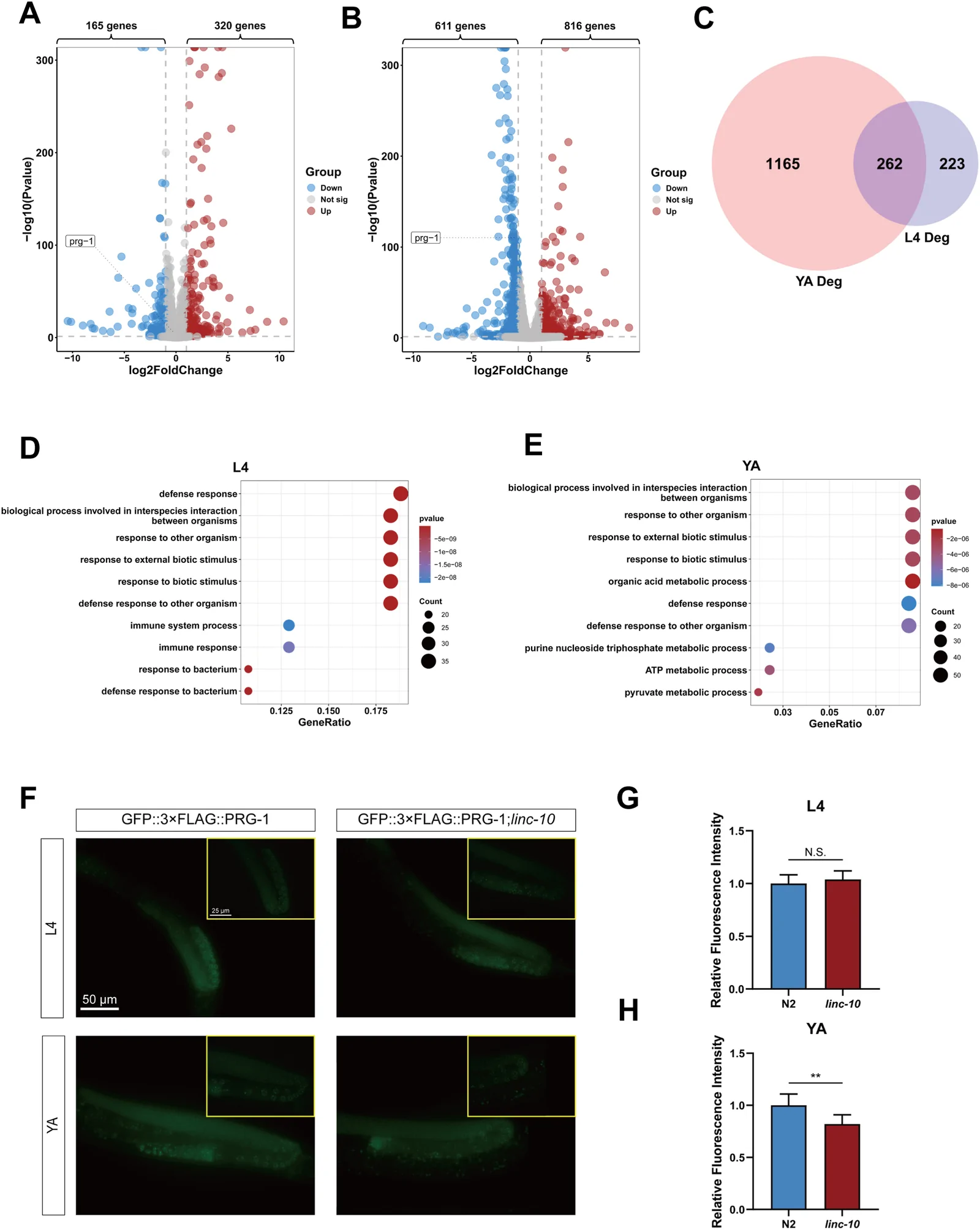
Transcriptomic changes and reduced PRG-1 expression in *linc-10* knockout *C. elegans*. (A) Volcano plot illustrating differential gene expression in *linc-10* knockout worms at the L4 larval stage. (B) Volcano plot showing differential gene expression in *linc-10* knockout young adults. (C) Venn diagram depicting 262 overlapping differentially expressed genes (DEGs) between the L4 and young adult stages. (D) Gene Ontology (GO) enrichment analysis of DEGs in L4-stage *linc-10* knockout worms. (E) GO enrichment analysis of DEGs in young adult *linc-10* knockout worms. (F) Representative fluorescence images of GFP*::*3×FLAG::PRG-1 in control and *linc-10* knockout backgrounds at the L4 and young adult stages. (G) Quantification of GFP fluorescence intensity at the L4 stage. (H) Quantification of GFP fluorescence intensity at the young adult stage. Bars represent means ± SD from three independent biological replicates, with 10 worms quantified per replicate. N.S., not significant; P < 0.01 (**). Differentially expressed genes shown in panels A–E were selected using *P* value < 0.05 and |log2FoldChange| ≥ 1.

### 3.4 Deletion of *linc-10* increases susceptibility to pathogen infection and environmental stress

We focused on the relationship between dysregulated gene expression and the stress-like phenotypes observed in *linc-10* KO worms. Additionally, the most prominent transcriptomic changes in the mutants involved genes related to innate immunity, pointing to a potential alteration in susceptibility to pathogens and stress. Based on this observation, we examined whether *linc-10* KO worms show differential sensitivity to pathogenic or environmental stressors compared to wild-type animals. As a baseline, the lifespan of *linc-10* KO mutants fed on *E. coli* OP50 did not show a significant difference compared to wild-type controls at 20 °C (Fig. 4A). In contrast, the mutants showed increased susceptibility to pathogens, as demonstrated in a *C. elegans* survival assay using *P. aeruginosa* PA14 (Fig. 4B). These data indicate that *linc-10* deletion is associated with increased susceptibility to PA14 infection. Furthermore, when exposed to heat and oxidative stress, *linc-10* KO worms also exhibited increased sensitivity compared to wild-type animals (Fig. 4C and D). The source data for lifespan, PA14 infection, heat stress, and oxidative stress assays are provided in Table S8. Taken together, the loss of *linc-10* in *C. elegans* resulted in heightened susceptibility to both pathogens and multiple forms of environmental stress.

**Fig. 4:**
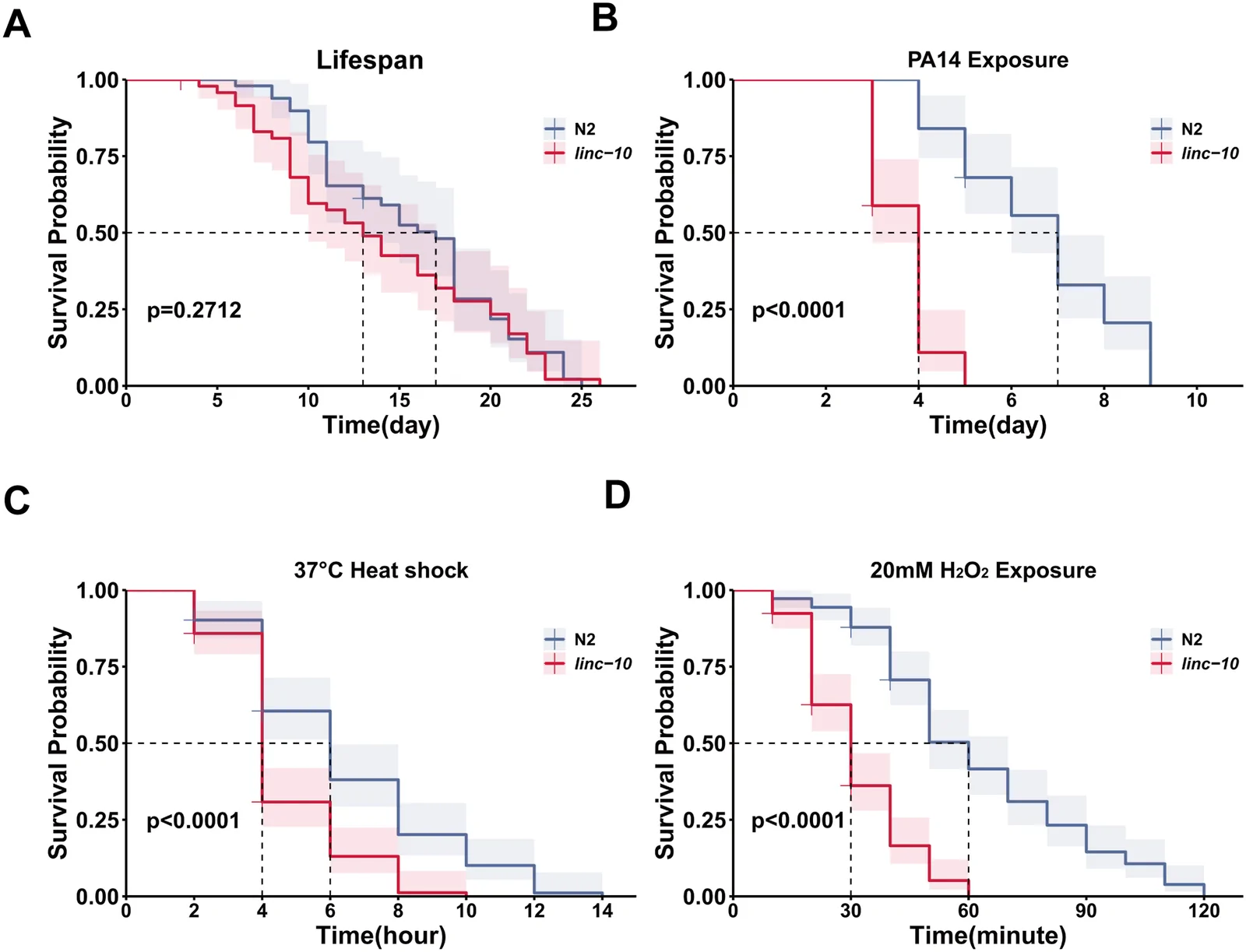
*linc-10* knockout increases sensitivity to *Pseudomonas aeruginosa* PA14 infection, heat stress, and oxidative stress in *C. elegans*. (A) Survival curves of *linc-10* KO worms and wild-type N2 controls at 20 °C. (B) *linc-10* KO worms exhibit increased susceptibility to *Pseudomonas aeruginosa* PA14 infection compared to wild-type N2. (C) *linc-10* KO worms are more sensitive to thermal stress at 37 °C. (D) *linc-10* KO worms showed increased sensitivity to 20 mM hydrogen peroxide (H_2_O_2_) exposure compared with N2. Each assay was performed in three independent biological replicates. The total numbers of animals analyzed in (A-D) were 101, 101, 184, and 212. Survival curves were analyzed using Kaplan–Meier analysis and compared by the log-rank test.

### 3.5 Changes in small RNAs following *linc-10* deletion

We further investigated whether *linc-10* deletion was associated with changes in small RNA expression. Small RNA sequencing revealed differential expression of both piRNAs and miRNAs in *linc-10* KO worms compared with wild-type animals. At the L4 stage, 29 piRNAs were differentially expressed, including 7 downregulated and 22 upregulated (Fig. 5A). At the young adult stage, 7 piRNAs were differentially expressed, all of which were upregulated (Fig. 5B). In animals, piRNAs mediate RNA silencing by guiding Piwi proteins via the RNA-induced silencing complex (RISC) [35]. In nematodes, this pathway regulates germline stem cell homeostasis, epigenetic processes, and innate immunity [40]. We identified the target mRNAs of these differentially expressed piRNAs using miRanda (Fig. 5C). Among these, 12 overlapped with the DEGs identified in our RNA-seq analysis of protein-coding genes (Fig. 5D). In addition, at the L4 stage, 8 miRNAs were differentially expressed, with 2 downregulated and 6 upregulated (Fig. 5E). In young adults, 8 miRNAs were differentially expressed, comprising 4 downregulated and 4 upregulated (Fig. 5F). Compared with piRNAs, which mainly act in germ cells, miRNAs function across diverse tissues and regulate post-transcriptional gene expression in *C. elegans*. We also identified the target mRNAs of the differentially expressed miRNAs, among which 15 overlapped with the DEGs identified in our RNA-seq dataset (Fig. 5G and H). In conclusion, *linc-10* deletion is associated with alterations in specific piRNAs and miRNAs, some of which may be linked to the mRNA expression changes observed in the mutants.

**Fig. 5:**
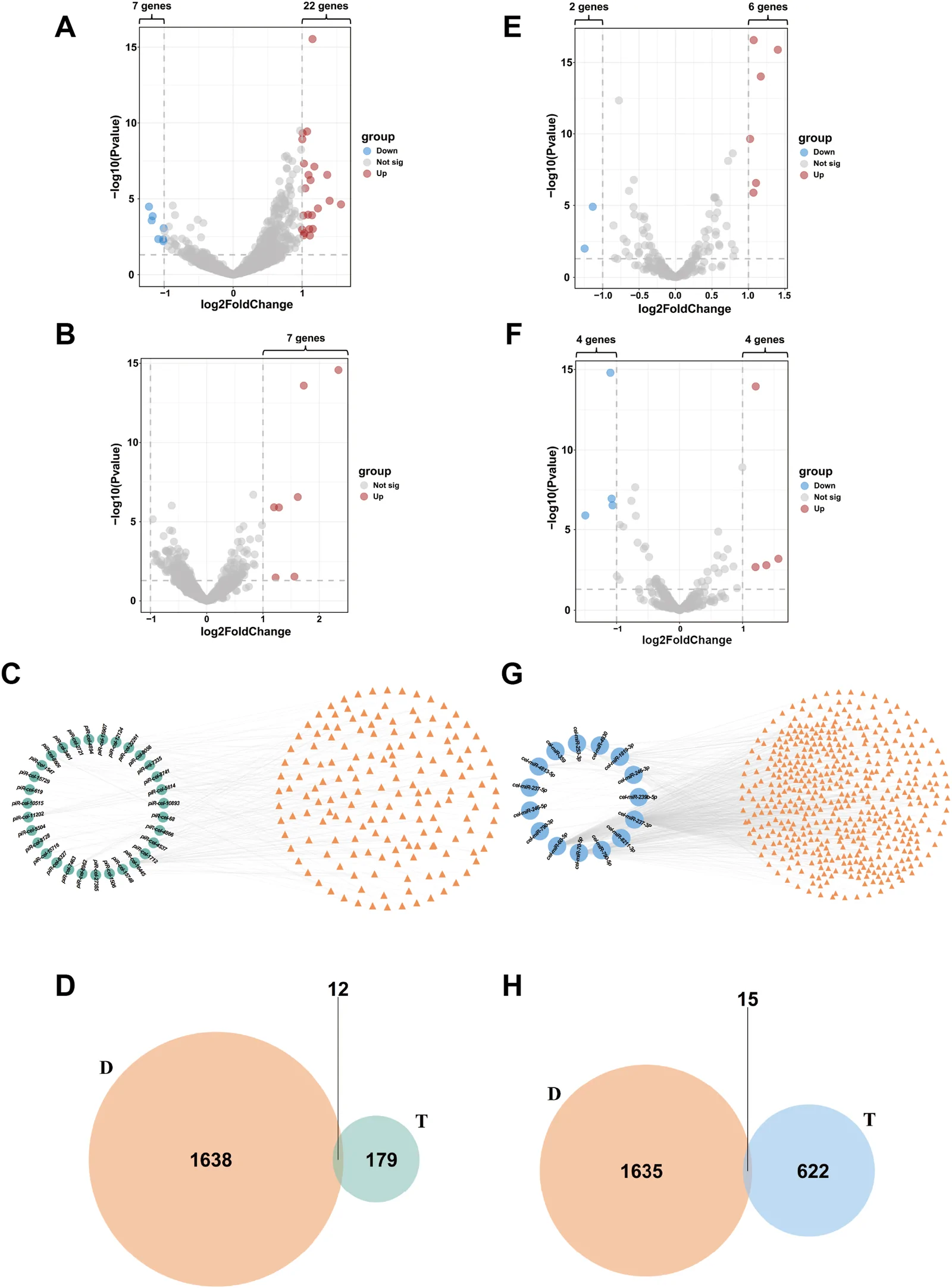
*linc-10* knockout alters small RNA expression. (A) Volcano plot showing differential expression of piRNAs in *linc-10* knockout worms at the L4 larval stage. (B) Differential expression of piRNAs in *linc-10* knockout young adults. (C) Predicted target genes of the dysregulated piRNAs. (D) Venn diagram showing the overlap between predicted piRNA targets and mRNA differentially expressed genes (DEGs) identified in this study. (E) Volcano plot showing differential expression of miRNAs in *linc-10* knockout worms at the L4 larval stage. (F) Differential expression of miRNAs in *linc-10* knockout young adults. (G) Predicted target genes of the dysregulated miRNAs. (H) Venn diagram showing the overlap between predicted miRNA targets and mRNA DEGs identified in this study. Differentially expressed piRNAs, miRNAs, and mRNAs used in these analyses were selected using *P* value < 0.05 and |log2FoldChange| ≥ 1. In panels D and H, D denotes mRNA DEGs and T denotes predicted target genes.

### 3.6 Rescue analysis supports the functional relevance of *linc-10*

To further assess whether the observed phenotypes were associated with the loss of *linc-10*, we generated a rescue strain carrying the *Plinc-10::linc-10* transgene in the *linc-10* mutant background. Successful expression of *linc-10* in the rescue strain was confirmed by qRT-PCR (Fig. S5). We first examined reproductive and developmental phenotypes. In the rescue strain, sterile animals were no longer observed under the present assay conditions, whereas the elevated male ratio was not significantly reduced compared with the *linc-10* mutant and remained higher than that of N2 animals (Fig. 6A). We next analyzed brood size. Compared with *linc-10* mutants, the rescue strain showed a significant increase in brood size, although the value remained lower than that of wild-type N2 animals (Fig. 6B). This result indicates that *Plinc-10::linc-10* partially rescued the reduced brood size phenotype. Consistently, larval growth analysis showed that the developmental delay observed in *linc-10* mutants was largely restored in the rescue strain, with no significant difference in body length between the rescue strain and N2 at any examined time point (Fig. 6C). The stress-sensitive phenotypes of *linc-10* mutants were also partially alleviated in the rescue strain under *P. aeruginosa* PA14 infection, heat stress, and H_2_O_2_-induced oxidative stress, although survival was not fully restored to the wild-type level (Fig. 6D–F). Together, these results indicate that reintroduction of *linc-10* rescued or partially rescued several reproductive, developmental, and stress-sensitive phenotypes of *linc-10* mutants, supporting the functional relevance of the *linc-10* transcript. The source data for the rescue experiments are provided in Table S9.

**Fig. 6:**
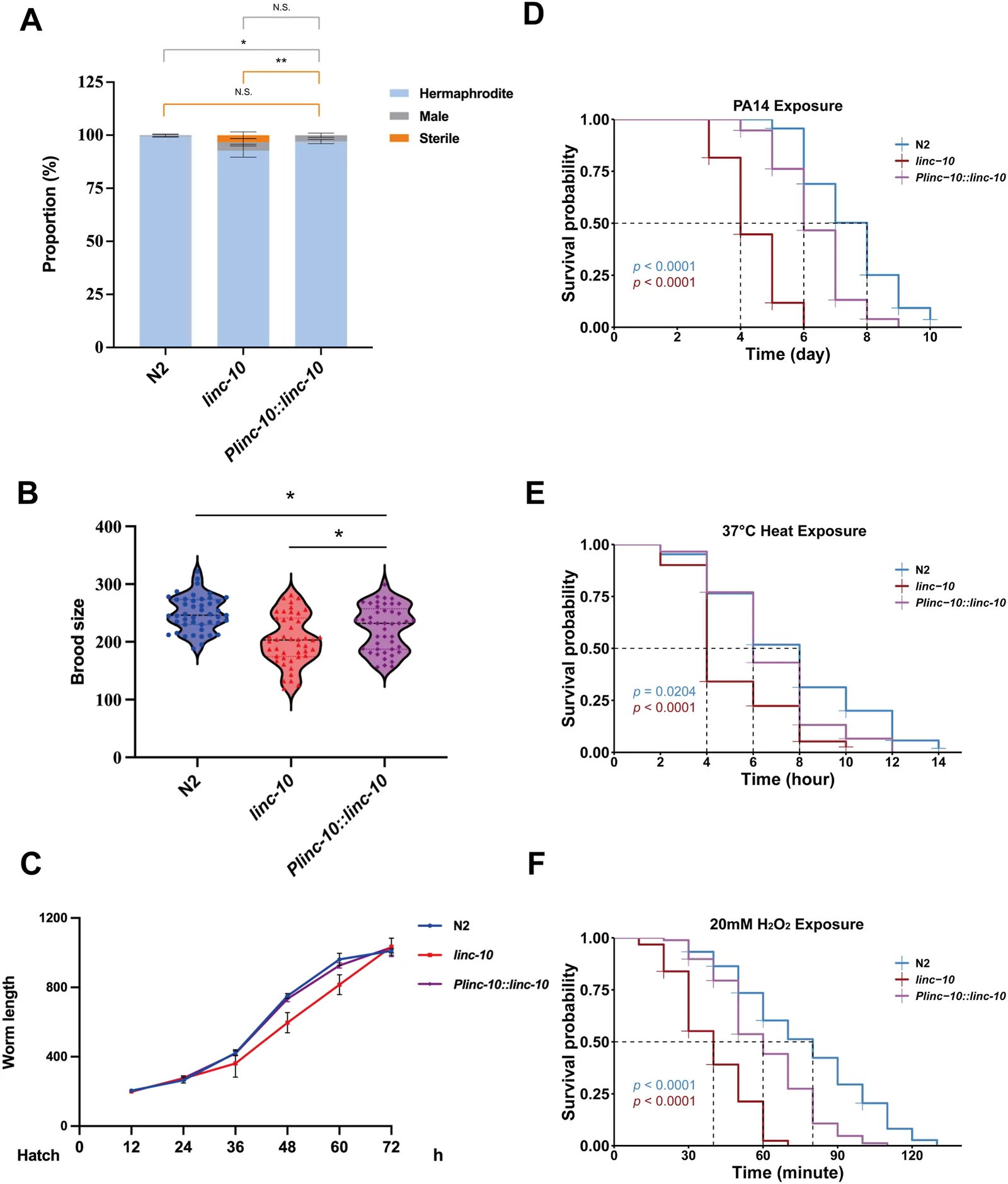
*Plinc-10::linc-10* transgene partially rescues the phenotypic defects of *linc-10* mutants. (A) Reintroduction of *linc-10* rescued the sterility phenotype but did not restore the increased male ratio to the wild-type level. Each independent experiment included 100 worms, and three independent experiments were performed. (B) The reduced brood size of *linc-10* mutants was partially restored by the *Plinc-10::linc-10* rescue transgene. Fifteen worms were scored in each independent experiment, and three independent experiments were performed. (C) Larval growth delay in *linc-10* mutants was largely restored by the *Plinc-10::linc-10 transgene*. Body length (μm) was measured every 12 h after hatching. Ten worms were measured per time point in each independent experiment, and three independent experiments were performed. (D) The increased susceptibility of linc-10 mutants to *P. aeruginosa* PA14 infection was partially rescued by the *Plinc-10::linc-10* transgene. Three independent experiments were performed, with a total of 276 worms analyzed. (E) The heat-stress sensitivity of *linc-10* mutants was partially rescued by the *Plinc-10::linc-10* transgene at 37 °C. Three independent experiments were performed, with a total of 263 worms analyzed. (F) The oxidative-stress sensitivity of *linc-10* mutants was partially rescued by the *Plinc-10::linc-10* transgene following exposure to H_2_O_2_. Three independent experiments were performed, with a total of 270 worms analyzed. Bars represent means ± SD from three independent biological replicates. N.S., not significant; P < 0.05 (*), P < 0.01 (**), determined by Fisher’s exact test in (A), unpaired *t*-test in (B) and (C), and log-rank test in (D–F).

## 4 Discussion

Noncoding RNAs play diverse and significant roles in organisms, as both their transcripts and the transcription processes are involved in the regulation of gene expression [41], [42]. In general, the regulatory targets of lncRNAs can be located on the genome either in their vicinity (i.e., cis-acting) or away from the lncRNA gene locus (i.e., trans-acting) [1]. In trans-regulation, lncRNAs control distal gene expression by interacting with promoters, enhancers, or associated proteins, thereby influencing chromatin state and the activity of RNA polymerase [43]. In cis-regulation, lncRNAs may function as regulatory transcripts directly affecting nearby gene expression, or their transcription might not result in a functional entity, where the processes of transcription or splicing are critical for regulation [44]. Additionally, lncRNAs can act as endogenous competing RNAs (ceRNAs) for microRNAs, titrating their availability, or serve as key cellular components with distinct regulatory roles [1]. These diverse functions underlie their important roles in both development and disease [2]. However, lncRNAs with detectable or even substantial roles at the cellular or tissue level do not necessarily produce overt organismal phenotypes when disrupted [4]. Therefore, animal models remain essential for evaluating whether and how lncRNAs contribute to physiological functions in vivo [4].

In this study, we systematically investigated the function and potential regulatory mechanisms of a long intergenic noncoding RNA, whose deletion results in an aberrant phenotype in *C. elegans*. Using a CRISPR knockout strain, we found that the deletion of *linc-10* caused the nematodes to exhibit a phenotype similar to that induced by stress or infection. The KO worms showed a lower number of offspring, while a small number of individuals exhibited sterility, and an increased proportion of males was observed (Fig. 1A–D). The expression patterns of *C. elegans linc-10* have been reported in several previous studies [8], [19], [39]. To further explore the expression patterns, we constructed a GFP reporter strain using CRISPR gene editing and detected GFP signals linked to the *linc-10* promoter. Briefly, the GFP signal was weak or barely distinguishable from intestinal autofluorescence during classical developmental stages, whereas clear reporter activity was observed in the male germline (Fig. 2A–D). These observations are consistent with the low overall expression of *linc-10* and suggest that *linc-10* may be associated with reproductive processes, particularly in males.

Next, we sought to identify molecular changes associated with the stress-like phenotype resulting from *linc-10* deletion. RNA sequencing revealed broad changes in protein-coding gene expression in *linc-10* KO worms, with innate immunity-related genes among the most prominently affected groups (Fig. 3A–E). Among the dysregulated genes, *prg-1* was reduced in young adult mutants, and this decrease was further supported by qRT-PCR and GFP reporter analyses (Fig. 3F–H). Given the established roles of PRG-1 in the piRNA pathway, germline homeostasis, and pathogen-related responses [32], [45], reduced PRG-1 expression may represent one molecular consequence of *linc-10* loss that is relevant to the reproductive and stress-related phenotypes. Consistent with the transcriptomic changes, *linc-10* KO mutants showed increased sensitivity to *P. aeruginosa* PA14 infection, heat stress, and oxidative stress, whereas their lifespan under standard culture conditions was not significantly altered (Fig. 4A–D). Small RNA sequencing further revealed altered piRNA and miRNA profiles following *linc-10* deletion, suggesting that loss of *linc-10* may also affect small RNA-associated regulatory networks (Fig. 5A–H). Importantly, introduction of a *Plinc-10::linc-10* rescue transgene rescued or partially rescued several phenotypic abnormalities of *linc-10* mutants, including sterility, developmental delay, reduced brood size, and stress-sensitive phenotypes (Fig. 6). Although the elevated male ratio was not restored and the rescue effects were incomplete, these results support the functional relevance of the *linc-10* transcript and strengthen the link between *linc-10* loss and the observed phenotypes. The rescue of multiple phenotypes by reintroduction of *linc-10* also argues against the possibility that the observed abnormalities arise solely from unintended effects associated with genome editing. Together, these findings indicate that *linc-10* deletion perturbs immune/stress-responsive gene expression and is accompanied by changes in PRG-1 expression and small RNA profiles, providing a molecular context for the observed stress-sensitive and reproductive phenotypes.

Due to their low expression levels and complex tissue and species specificity, the functions and mechanisms of lncRNAs are not as well understood as those of protein-coding genes [1]. Although lncRNAs are involved in the regulation of gene expression through various mechanisms at the cellular level, studies on *C. elegans* demonstrated that long intergenic noncoding RNAs have no major effects on survival or fertility [8], [17], [20]. These lincRNAs may nevertheless have regulatory roles in reproduction and responses to environmental changes, with some reproductive functions becoming more apparent when multiple lincRNAs are disrupted [21], [46]. Here, our findings add *linc-10* to the small group of *C. elegans* lincRNAs whose loss produces detectable but non-lethal organismal phenotypes, particularly under pathogen and stress conditions. In our previous work, *linc-10* deletion was associated with altered offspring number in *C. elegans* [8]. The present study extends this observation by showing that loss of *linc-10* is associated with a broader set of mild but detectable phenotypes, including increased susceptibility to pathogenic infection and environmental stressors. These phenotypes were consistent with the changes observed in innate immunity-related gene expression in the mutants. In addition, deletion of *linc-10* influenced the expression of PIWI Argonaute homolog PRG-1. Whether this change reflects a direct regulatory effect of *linc-10*, an indirect consequence of altered germline physiology, or a compensatory response to the mutant state remains to be determined. Given the diverse modes by which lncRNAs can influence gene expression [47], [48], [49], future studies will be needed to determine how *linc-10* loss leads to reduced PRG-1 expression. Together with the dysregulation of protein-coding genes involved in immune and stress responses, the altered piRNA and miRNA profiles suggest that *linc-10* deletion is accompanied by broader changes in gene-regulatory networks; notably, the miRNA changes may also be biologically relevant given the smaller number of annotated miRNAs in *C. elegans*. These changes may be relevant to the reproductive defects and stress-sensitive phenotypes observed in the mutants, although their relative contributions remain unresolved. Because the rescue construct was inserted into a neutral intergenic site rather than restoring the endogenous locus, the partial rescue suggests that *linc-10* can function, at least in part, as a trans-acting transcript. This observation is consistent with the possibility that *linc-10* contributes to phenotype regulation through effects on gene expression, although locus-dependent or genomic-context-dependent effects cannot be excluded [47], [50].

However, several questions remain unresolved. Although transcriptomic and small RNA analyses identified multiple molecular changes associated with *linc-10* deletion, the present data do not distinguish whether these alterations represent primary regulatory events or secondary consequences of the mutant state. Reduced PRG-1 expression and altered piRNA and miRNA profiles were observed in *linc-10* mutants, but their mechanistic relationships remain unclear. Although a subset of predicted piRNA and miRNA targets overlapped with the mRNA DEGs identified in this study (Fig. 5D, H), the biological significance of this overlap remains to be further examined. We did not determine whether this overlap exceeds random expectation or whether the direction of mRNA changes is fully consistent with the predicted regulatory relationships. Therefore, these overlapping genes should be viewed as candidate associations that may help prioritize future validation rather than as definitive evidence of small RNA-mediated regulation. The predicted piRNA targets should therefore be interpreted as candidate associations rather than evidence of direct germline regulation, which will require future experimental validation. In addition, the relatively limited overlap between differentially expressed genes and predicted small RNA targets suggests that additional regulatory pathways may contribute to the observed phenotypes. Further genetic and molecular studies are needed to define the causal mechanisms linking *linc-10* loss to reproductive and stress-related phenotypes. In summary, our findings characterize the organismal phenotypes associated with *linc-10* deletion in *C. elegans* and provide clues to the regulatory changes that may underlie its roles in reproductive fitness and stress resilience.

## 5 Conclusion

In this study, we demonstrated that loss of *linc-10* in *C. elegans* results in mild but notable phenotypes, including reduced brood size, altered developmental trajectories, the occurrence of a small proportion of sterile individuals, and an increased male ratio. Reporter assays revealed that *linc-10* is predominantly expressed in the male gonad. Functional analyses further showed that *linc-10* knockout is associated with altered expression of innate immunity-related genes and increased susceptibility to pathogenic infection and environmental stressors. At the molecular level, *linc-10* deletion was associated with altered piRNA and miRNA profiles. Introduction of a *Plinc-10::linc-10* rescue transgene into a neutral intergenic site partially rescued several phenotypic defects of *linc-10* mutants. Collectively, these findings suggest that *linc-10* contributes to reproductive fitness, immune homeostasis, and stress resilience in *C. elegans.*

## CRediT authorship contribution statement

**Chenxi Zhou:** Writing – original draft, Visualization, Investigation, Formal analysis, Data curation. **Xiaoman Sun:** Writing – review & editing, Validation, Investigation. **Ruikun Wu:** Validation, Methodology, Investigation. **Zifan Wu:** Validation, Data curation. **He Chen:** Writing – original draft, Supervision, Project administration, Funding acquisition, Data curation, Conceptualization.

## Data availability

All raw sequencing data supporting the findings of this study, including transcriptome sequencing and small RNA sequencing data from *Caenorhabditis elegans* wild-type N2 and *linc-10* knockout strains at the L4 and young adult stages, are available in the NCBI Sequence Read Archive (SRA) under the BioProject accession number PRJNA1478352.

## Ethics approval and consent to participate

*C. elegans* is exempt from ethics approval.

## Declaration of generative AI and AI-assisted technologies in the writing process

During the preparation of this work the authors used ChatGPT (OpenAI) in order to improve the clarity of English expression. After using this tool, the authors reviewed and edited the content as needed and take full responsibility for the content of the published article.

## Funding

This work was supported by the 10.13039/501100001809National Natural Science Foundation of China (No. 31900442) and the Research Start-up Fund of 10.13039/501100002729Anhui University.

## Declaration of competing interest

The authors declare that they have no known competing financial interests or personal relationships that could have appeared to influence the work reported in this paper.

## Figures and Tables

**figs1:**
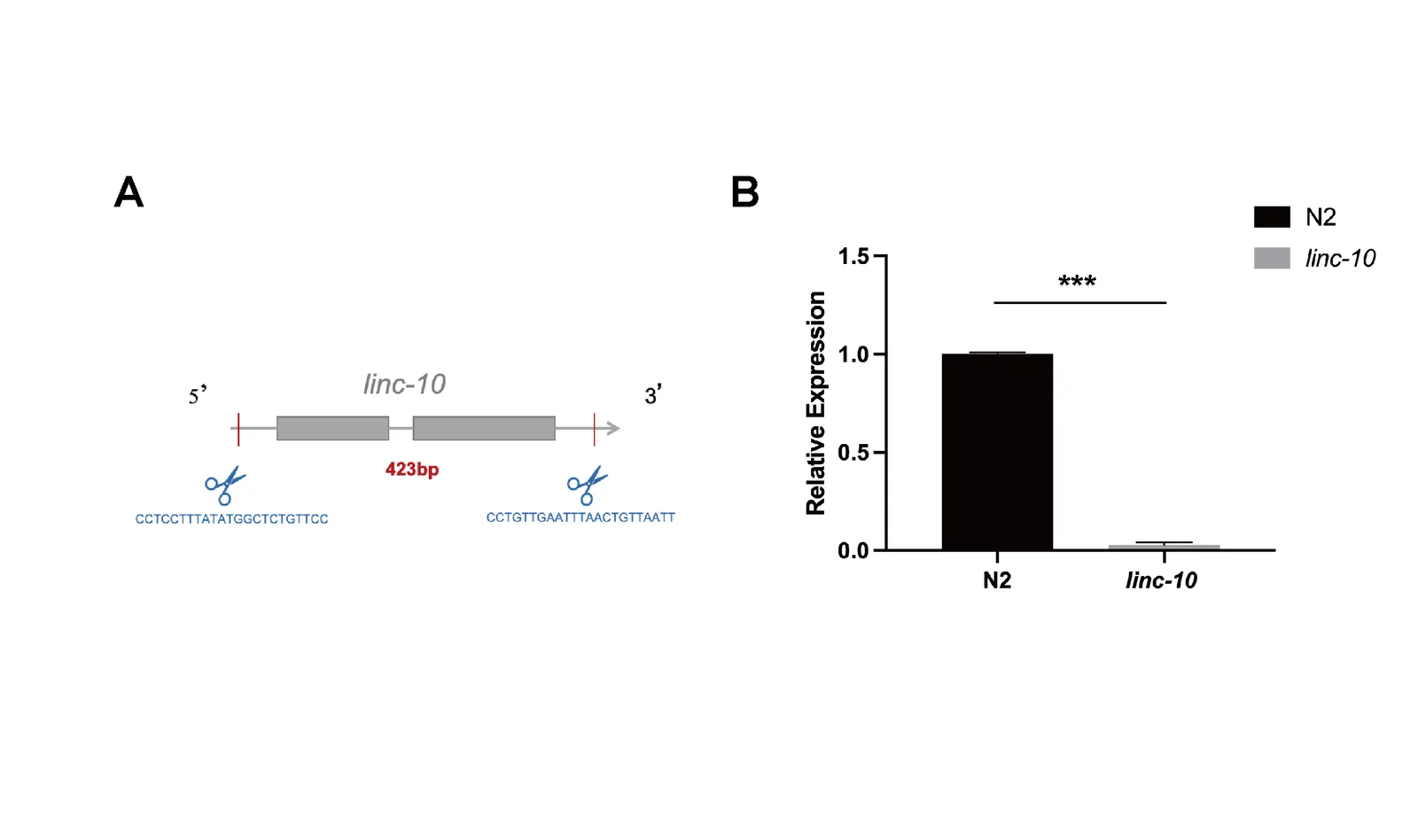


**figs2:**
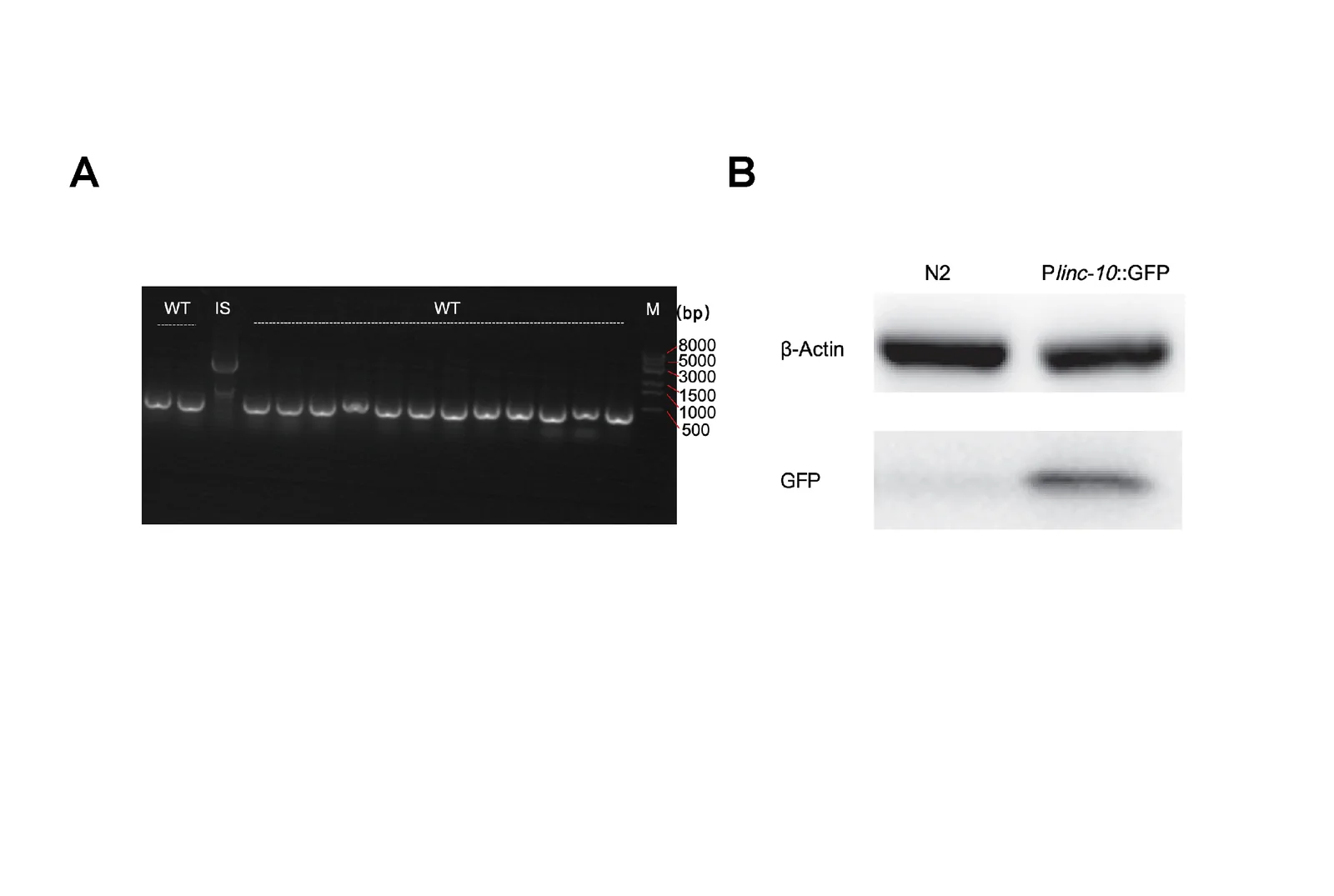


**figs3:**
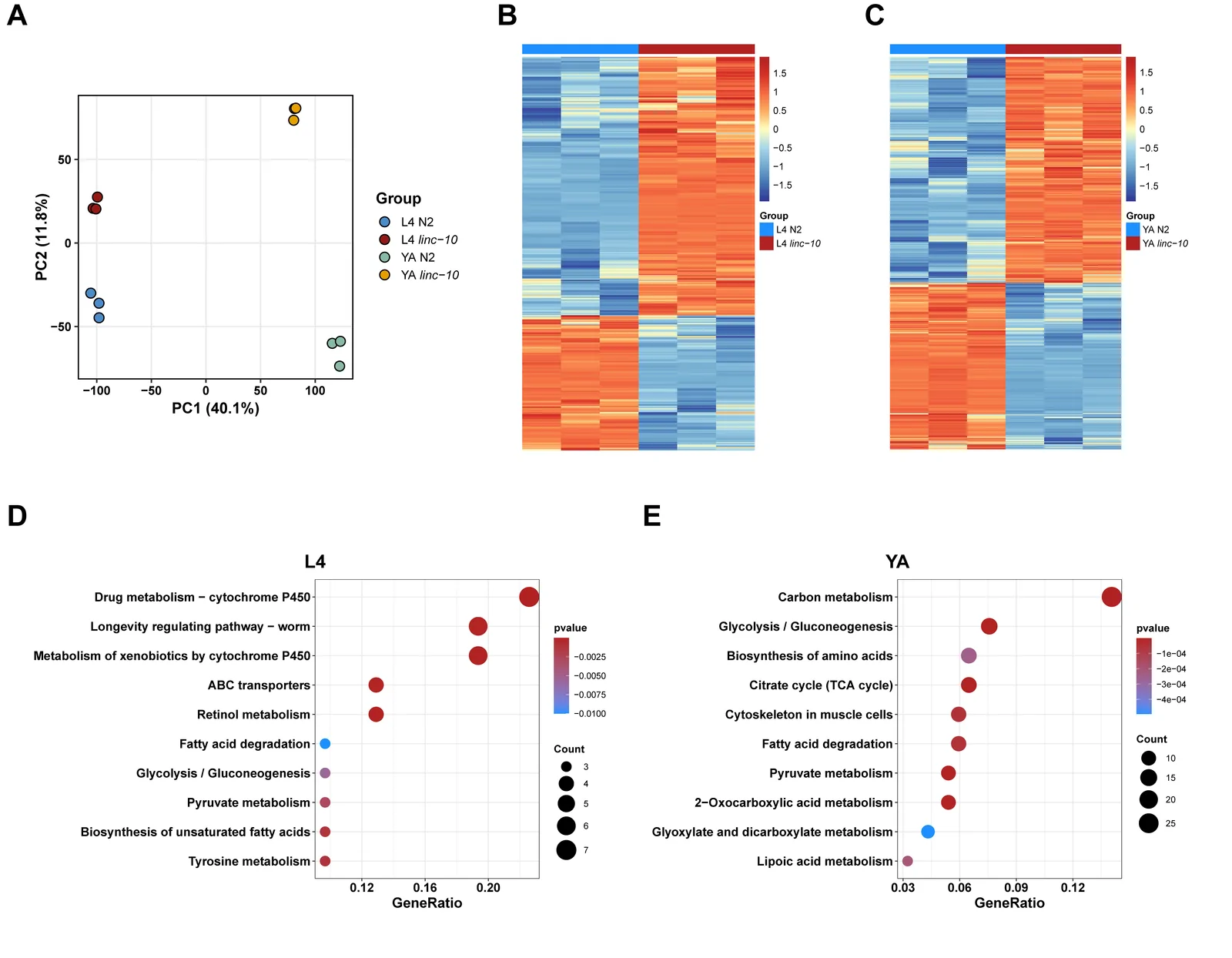


**figs4:**
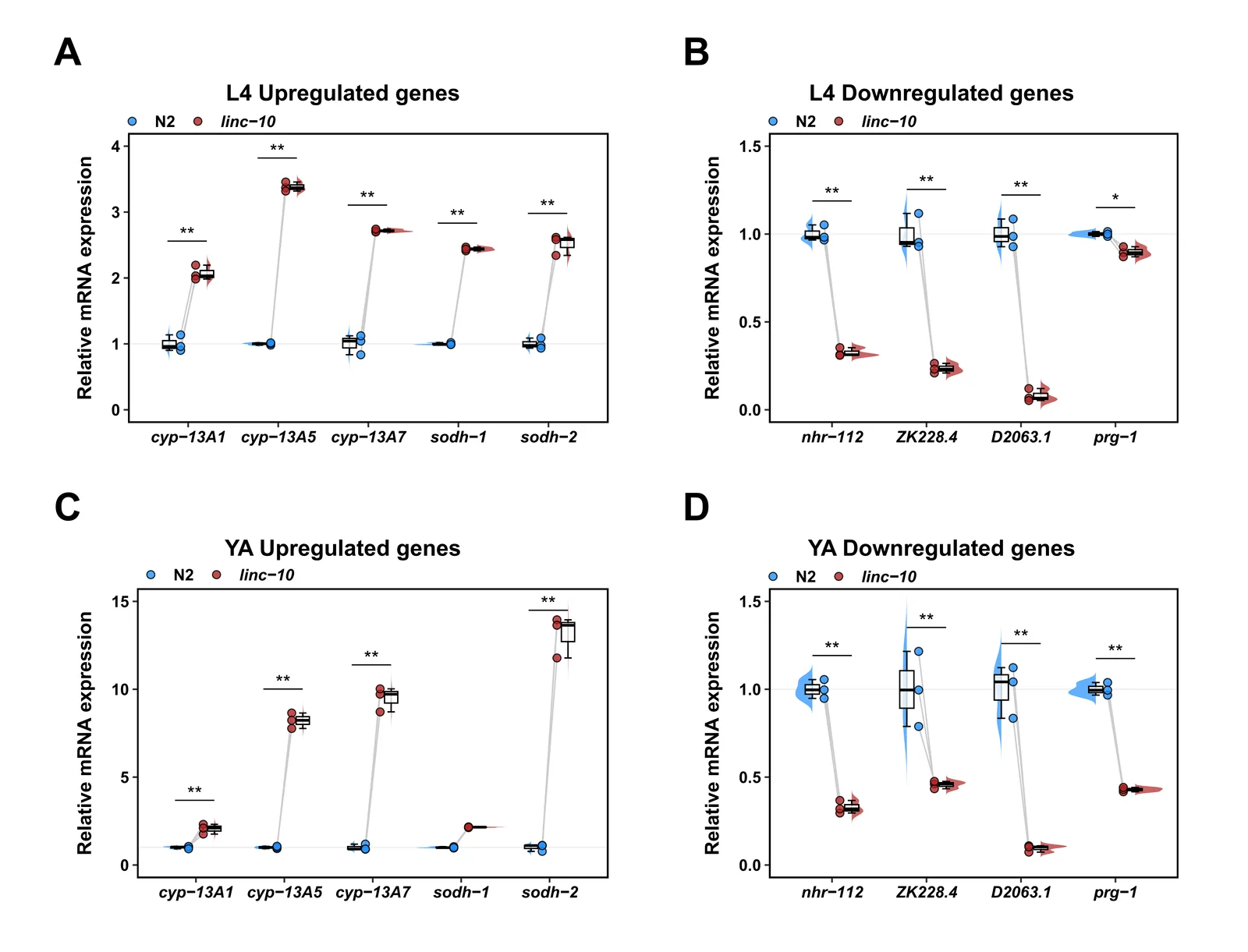


**figs5:**
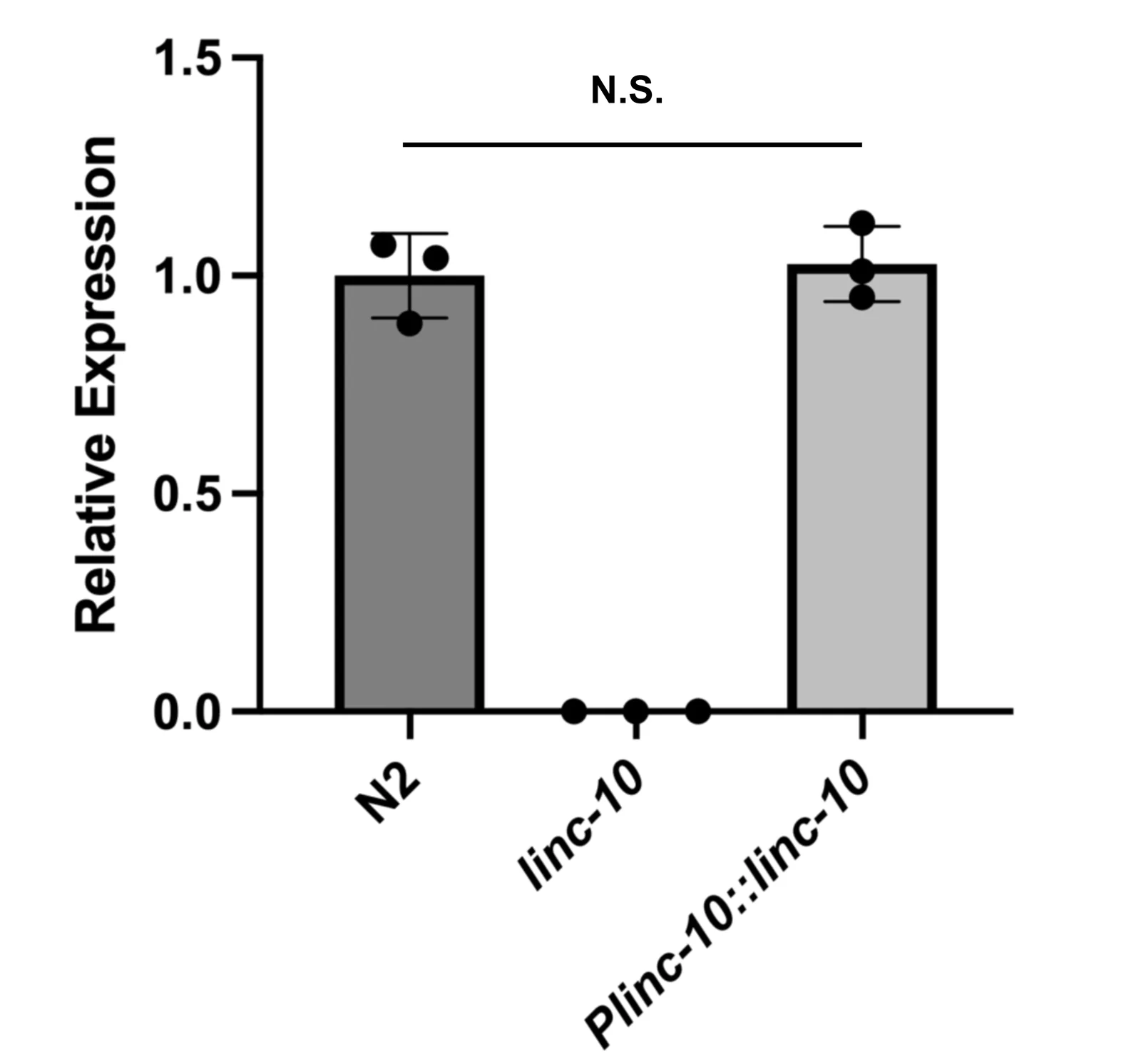


**figs6:**