# Supplementary material for: Discernible phenotypic alterations following linc-10 knockout in Caenorhabditis elegans
Source: Noncoding RNA Res. 2026 Nov 4;21:91–103. doi: 10.1016/j.ncrna.2026.10.004 (PMC13756530; doi:10.1016/j.ncrna.2026.10.004)
Supplement: Supplementary file 1 [file mmc11.docx]

Figure S1. Characterization of *linc-10* Knockout *C. elegans*

(A) Construction of *C. elegans linc-10* knockout mutants via CRISPR/Cas9-mediated genome editing.

(B) Quantitative reverse transcription PCR (qRT-PCR) analysis confirming loss of *linc-10* expression in the mutant strain.

Bars represent means ± SD. P < 0.001 (***).

Figure S2**.** Validation of *Plinc-10::GFP* reporter strain construction.

(A) Genotyping of transgenic C. elegans expressing *Plinc-10::GFP*. PCR primers were designed in the upstream region of the *linc-1* gene, where the transgene was inserted. Agarose gel electrophoresis shows a 348 bp band in wild-type (WT) animals and a 3233 bp band in insertion strain (IS) animals carrying the GFP transgene. M: DNA marker.

(B) Western blot analysis showing GFP protein expression in the reporter strain.

Figure S3. Transcriptomic analysis of *linc-10* knockout worms.

(A) Principal component analysis (PCA) of transcriptomic profiles from wild-type N2 and *linc-10* knockout worms at the L4 larval and young adult stages.

(B) Heatmap showing expression patterns of differentially expressed genes (DEGs) in *linc-10* knockout worms at the L4 larval stage.

(C) Heatmap showing expression patterns of DEGs in *linc-10* knockout worms at the young adult stage.

(D) KEGG pathway enrichment analysis of DEGs in L4-stage *linc-10* knockout worms.

(E) KEGG pathway enrichment analysis of DEGs in young adult *linc-10* knockout worms.

Figure S4. qRT-PCR validation of representative differentially expressed genes in *linc-10* knockout worms.

(A) qRT-PCR analysis of representative upregulated genes in *linc-10* knockout worms at the L4 larval stage.

(B) qRT-PCR analysis of representative downregulated genes, including prg-1, in *linc-10* knockout worms at the L4 larval stage.

(C) qRT-PCR analysis of representative upregulated genes in *linc-10* knockout worms at the young adult stage.

(D) qRT-PCR analysis of representative downregulated genes, including prg-1, in *linc-10* knockout worms at the young adult stage.

Relative expression levels were normalized to wild-type N2 controls. Bars represent means ± SD from three independent biological replicates. P < 0.05 (*), P < 0.01 (**).

Figure S5. Validation of *linc-10* expression in the rescue strain.

qRT-PCR analysis showing restored *linc-10* expression in *Plinc-10::linc-10* rescue worms. Relative expression levels were normalized to those in wild-type N2 controls. Bars represent means ± SD. N.S., not significant.
